# Effects of different simulated submarine escape depths by free ascent in animal models

**DOI:** 10.3389/fphys.2023.1107782

**Published:** 2023-01-27

**Authors:** Xiao Chen Bao, Nan Wang, Ji Xu, Jun Ma, Yi-Qun Fang

**Affiliations:** Department of Diving Medicine, Naval Medical Center, Shanghai, China

**Keywords:** decompression illness, coagulation, myocardial enzymes, pulmonary function, submarine escape, free ascnet

## Abstract

**Objective:** If a damaged submarine cannot be rescued in time, it is necessary to carry out a submarine escape by free ascent. Decompression illness is the greatest threat to the safety of submariners. The maximum depth at which a safe escape can be carried out is unknown. This study intends to explore the maximum safe escape depth by observing the effects of simulated submarine escape at different depths on animal models.

**Methods:** We evaluated pulmonary function indexes, blood gas values, blood cell counts, the myocardial enzyme spectrum, coagulation parameters, and proinflammatory cytokine levels in rats, electrocardiographic activity in rabbits after simulated 150-m, 200-m, 220-m, and 250-m submarine escape by free ascent.

**Results:** An escape depth of 150 m did not cause significant changes in the indicators. An escape depth of >200 m led to pulmonary ventilation and gas diffusion dysfunction, hypoxemia, myocardial ischemia, and activation of the fibrinolytic and inflammatory systems. The magnitudes of the changes in the indicators were proportional to escape depth.

**Conclusion:** An escape depth of 150 m in animal models is safe, whereas escape at > 200 m can be harmful.

## Introduction

If a submarine is unable to surface, the preferred option for the survivors is to await rescue within the distressed submarine (DISSUB). However, if environmental conditions within the DISSUB make waiting for rescue untenable then a submarine escape by free ascent is performed. The escape procedure involves rapid pressurization of one or two personnel at a time to the ambient sea pressure in the escape tower. When the tower pressure is equalized to the ambient sea pressure, the upper hatch opens, and the submariners ascend to the surface due to the buoyancy of the Submarine Escape Immersion Suit.

It is anticipated that decompression illness (DCI) limits the maximum depth at which escape can be achieved, and that the risk of pulmonary barotrauma limits the pressurization and ascent rates. The Royal Navy has done a lot in the development of modern submarine escape by free ascent. The testing of human subjects is reported up to real escapes by subjects from submarines lying at 180 m (Donald, 1979). However, the deep-diving capability of modern submarines means that the crew may need to escape at great depth. Can the maximum escape depth be further increased? Will a deeper escape depth have adverse effects on organs? We reported that the main manifestations of DCI induced in animal models by unsafe submarine escape are heart damage, acute pulmonary edema, coagulation and fibrinolysis system disorders (Bao et al., 2015) and subsequent activation of the inflammatory system (Wang et al., 2015a; Wang et al., 2015b; Wang et al., 2018). However, the effect of escape depth on these parameters is unclear.

Here, we evaluated the effects of escape depth on electrocardiographic activity, pulmonary function indexes, blood gas values, blood cell counts, the myocardial enzyme spectrum, coagulation parameters, and proinflammatory cytokine levels in rat lung tissue.

## Materials and methods

### Chemicals and reagents

Interleukin-6 (IL-6), IL-1β, and tumor necrosis factor-α (TNF-α) enzyme-linked immunosorbent assay (ELISA) kits were obtained from Elabscience Biotechnology (Wuhan, China). Acetonitrile, methanol, and water were of high-performance liquid chromatography grade (Thermo Fisher Scientific, Waltham, MA, USA).

### Animals

All studies with animals were approved by the Animal Care and Use Committee of Naval Medical Center. Adult 8–9-week-old male Sprague Dawley rats were used. In addition, six male New Zealand white rabbits weighing 3 kg were used to assess electrocardiographic activity. Animals were housed in cages and acclimatized to the housing facility for 1 week before experiments. Rats were randomly divided into the control group and 150-m, 200-m, 220-m, and 250-m escape groups (*n* = 10 per group). Rabbits were randomly divided into 150-m, 200-m, and 220-m escape groups (*n* = 2 per group).

### Escape protocol

**FIGURE 1:**
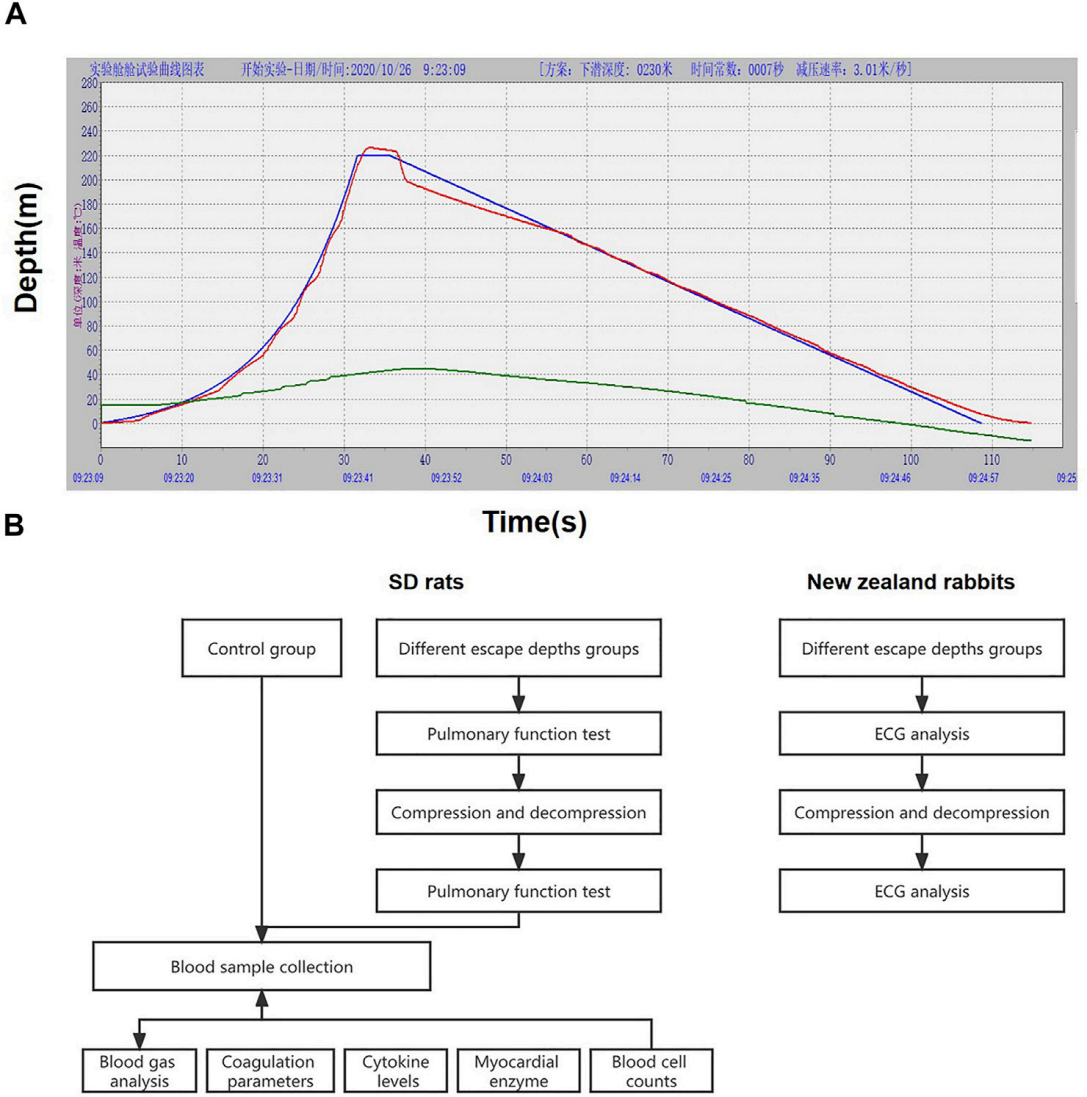
Pressure and decompression curves **(A)** and experimental flow chart **(B)**. Figure A is the 220 m escape curve, the green curve is the ideal pressure and decompression curve simulated by the computer, the red curve is the actual pressure and decompression curve, and the blue curve represents the temperature in the cabin.

For each escape group, animals were placed in the chamber. The pressurization and decompression procedure of the chamber was controlled by computer. Gas in the chamber was compressed to 150 m, 200 m, 220 m, and 250 m with 2^1/4^ index. Following a short hold at depth (4 s of bottom time), the chamber was decompressed to the surface at 3 m/s (0.03 MPa) (Figure 1).

### Sample collection

After decompression, rats were removed from the chamber and pulmonary function tests were performed. At 30 min after decompression, rats were anesthetized with 3% sodium pentobarbital, and blood samples were collected. The control rats were placed in the chamber without compression and decompression.

### Electrocardiography

Electrocardiography (ECG) was performed on rabbits before compression and after decompression. New Zealand rabbits were weighed, and 30 g/L sodium pentobarbital was slowly injected through the ear vein at a dose of 30 mg/kg. After anesthesia, the rabbit was placed supine and fixed on the operating table, and needle-shaped electrodes were inserted under the skin of the limbs. Three standard limb leads (I, II, and III) and three augmented limb leads (aVR, aVL, and aVF) were recorded. The calibration voltage was adjusted to 1 mV = 10 mm, the paper speed was 25 mm/s, and the electrocardiogram was recorded under quiet conditions.

### Non-invasive pulmonary function test

The pulmonary function of rats was tested by whole-body plethysmograph (Shanghai Tawang Intelligent Technology Co., Ltd., Shanghai, China). Awake rats were placed in four individual tracing boxes, the oxygen valve was opened, and the rats were accustomed to voluntary activities for 10 min. After adaptation, the basal inspiration time (Ti), expiration time (Te), peak inspiratory flow (PIF), peak expiratory flow (PEF), frequency (f), enhanced pause (Penh), midexpiratory flow (EF50), relaxation time (Tr), tidal volume, minute ventilation (MV), cumulative air volume (AV), difference between inspiratory/expiratory volume, and peak expiratory time ratio (Rpef) values were recorded over 3 min.

### Blood sampling and blood gas analysis

Blood samples were obtained after 30 min of observation. Arterial blood (0.5 mL) was collected from the femoral artery, fixed in heparin sodium (10 U/mL) to avoid coagulation, and analyzed using a portable blood gas analyzer (Abbott, IL, USA). Venous blood (6 mL) was taken from the right heart. Sample (1 mL) was injected into a vacutainer containing ethylene diamine tetraacetic acid as an anticoagulant (Becton, Dickinson and Company, Franklin Lakes, NJ, USA) and analyzed using a blood counter (Abbott). Also, a 1-mL sample was used for assays of creatine kinase (CK), lactate dehydrogenase (LDH), LDH1, aspartate transaminase (AST), α-hydroxybutyrate dehydrogenase (α-HBDH), and myocardial-specific isoenzyme of creatine kinase (CK-MB) activities.

### Coagulation parameters

Blood samples (2 mL) were dispensed into 3.2% sodium citrate. The prothrombin time (PT), activated partial thromboplastin time (aPTT), thrombin time (TT), D-dimer concentration, and fibrinogen (FIB) concentration were measured by a dry-hematology analyzer (COAG2NV, ERMA Inc., Tokyo, Japan).

### Quantification of proinflammatory cytokine levels

Venous blood (2 mL) was drawn into tubes lacking anticoagulant and placed at 4°C for 2 h. The samples were centrifuged at 1000 × *g* at 4°C for 20 min and the supernatant was stored at −80°C. The levels of IL-1β, TNF-α, and IL-6 were evaluated by ELISA (Elabscience Biotechnology Co., Ltd.) according to the manufacturer’s instructions.

### Statistical analysis

Statistical analysis was performed with Prism (GraphPad Software, La Jolla, CA, United States). Normally distributed data are presented as the means ± standard error of mean and non-normally distributed data as medians (minimum–maximum). Paired *t*-test and one-way analysis of variance (ANOVA) were performed to compare measurement data among groups. A *p*-value of < 0.05 was considered statistically significant.

## Results

### Incidence and symptoms of DCI

Rats in the 150-m escape group showed no symptoms, whereas those in the 200-m, 220-m, and 250-m escape groups had shortness of breath, laziness, and some had cyanotic symptoms. No other symptoms of DCI were observed.

### Effect of escape depth on ECG activity

**FIGURE 2:**
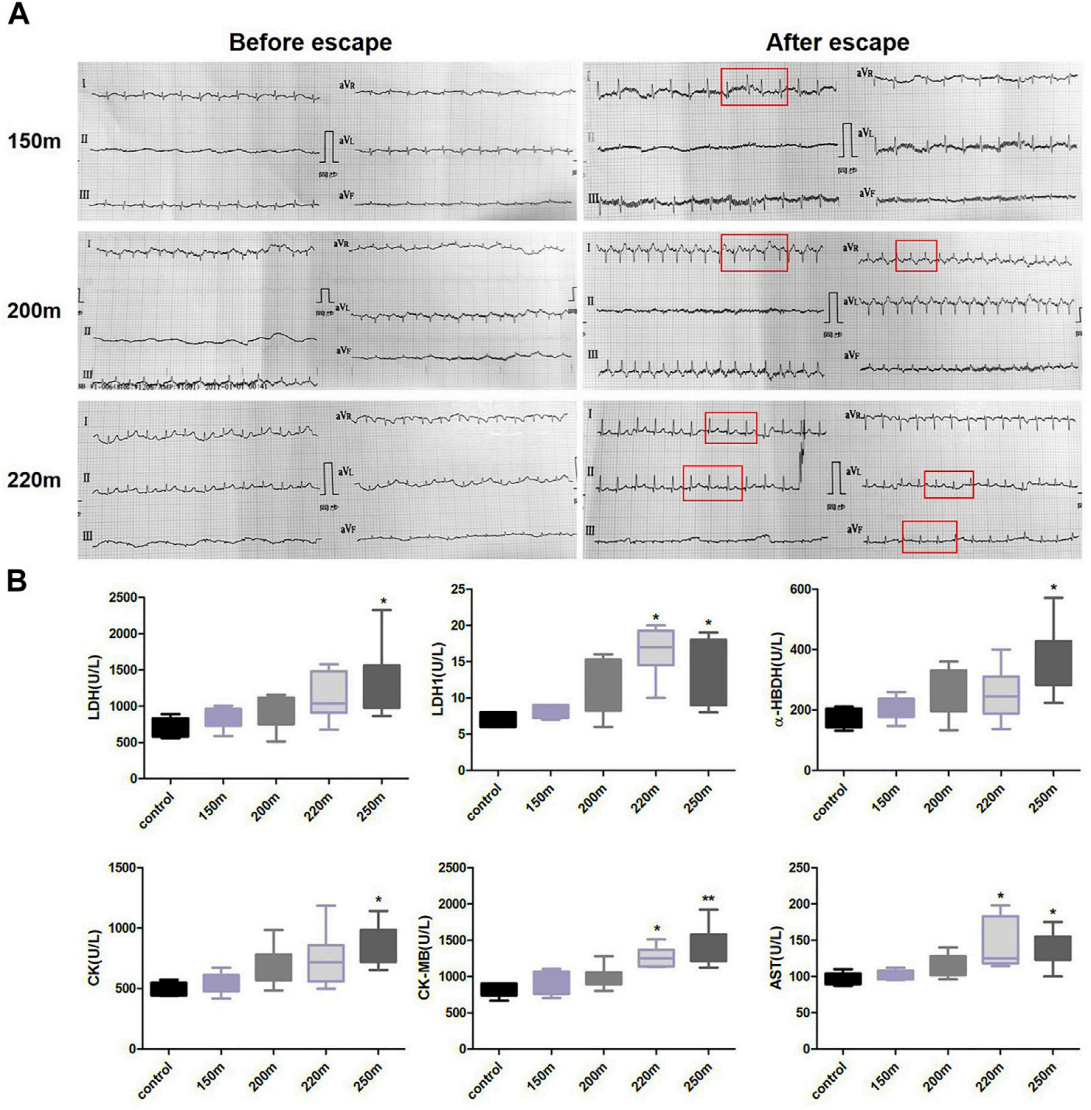
Effects of depth of simulated submarine tower escape on ECG activity **(A)** and myocardial enzyme spectrum **(B)**. Compared to before escape, increased heart rhythm and sinus arrhythmia were detected in the 150-m escape group. Rapid heart rate, peaked T waves, and Q wave appeared at a depth of 200 m. Pathological Q waves appeared at I, II, aVL, and aVF leads at a depth of 220 m. One-way ANOVA was performed based on the normality of the distribution by Kolmogorov–Smirnov test. Results are the minimum to maximum values (*n* = 5–7 per group). *p < 0.05, **p < 0.01 compared to the control.

The ECG results changed with increasing depth of escape (Figure 2A). The R wave at each escape depth increased, indicating ventricular high voltage, especially at 220 m. Compared with before escape, after 150-m escape, ECG showed increased heart rate and sinus arrhythmia; after 200-m escape, rapid heart rate and peaked T waves appeared, and Q wave appeared in aVR lead; after 220-m escape, the pathological Q wave appeared at leads I, II, aVL, and aVF.

### Effect of escape depth on the myocardial enzyme spectrum

Myocardial enzyme levels showed an increasing trend with escape depth (Figure 2B). Compared with the normal control group, the LDH, CK, and α-HBDH values of the 250-m escape group were significantly higher (LDH: 1,319 ± 331 U/L *vs*. 708 ± 56.88 U/L; *p* = 0.015; CK: 856.9 ± 65.28 U/L *vs*. 486.2 ± 26.26 U/L; *p* = 0.001; α-HBDH: 352.0 ± 44.25 U/L *vs*. 177.2 ± 14.87 U/L; *p* = 0.014). Moreover, the AST and CK-MB values of the 200-m and 250-m escape groups were significantly higher than the control group (AST: 141.3 ± 12.94 U/L, 135.4 ± 9.17 U/L *vs*. 96.40 ± 3.86 U/L; *p* < 0.05; CK-MB: 1,267 ± 57.02 U/L, 1,392 ± 106.5 U/L *vs*. 824.6 ± 42.94 U/L; *p* < 0.05).

### Effect of escape depth on pulmonary function

**FIGURE 3:**
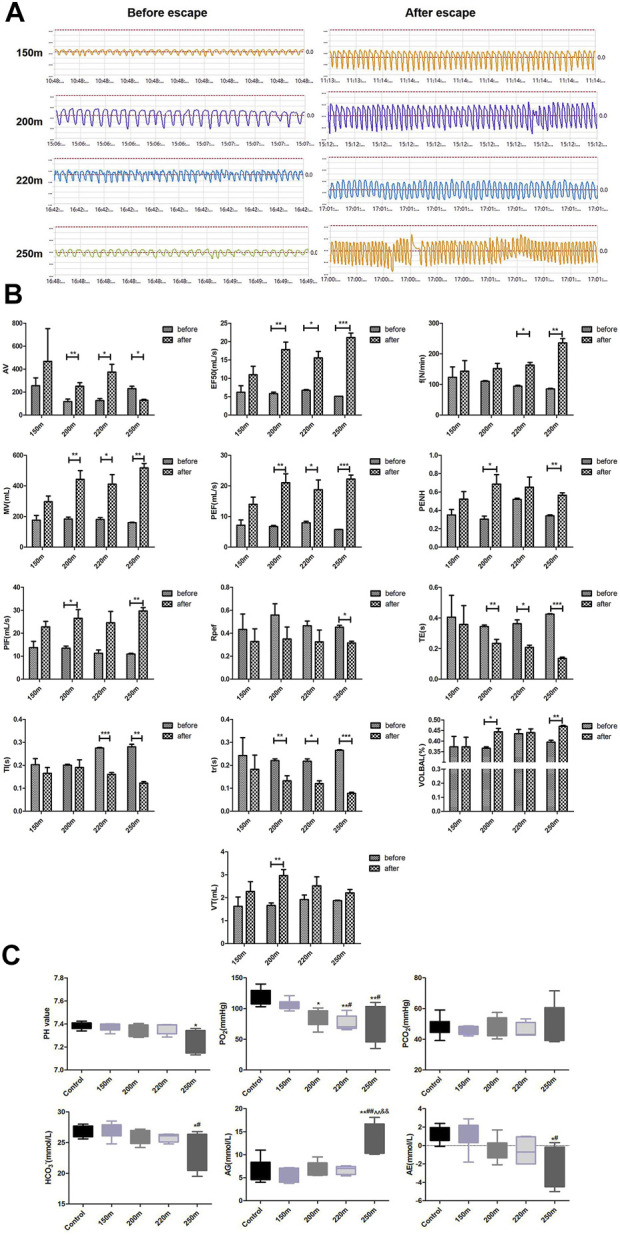
Effect of depth of simulated submarine tower escape on pulmonary function and acid–base status. **(A)** Respiration waveforms of rats before and after escape. **(B)** Lung function indexes before and after escape. Paired *t*-test was performed based on the normality of the distribution by Kolmogorov–Smirnov test. Results are minimum to maximum values (*n* = 4–6 per group). *p < 0.05, **p < 0.01, ***p < 0.001 compared to before escape. **(C)** Effect of depth of simulated submarine tower escape on acid–base status. One-way ANOVA was performed based on the normality of the distribution by Kolmogorov–Smirnov test. Results are the minimum to maximum values (*n* = 5–7 per group). *p < 0.05, **p < 0.01 compared to the control; ^#^ *p* < 0.05, ^##^ *p* < 0.01 compared to 150-m escape; ^^*p* < 0.01 compared to 200-m escape; ^&&^ *p* < 0.01 compared to 220-m escape.

The 150-m escape did not significantly influence pulmonary function indicators (Figures 3A, B). With increasing escape depth, f and MV increased significantly, indicating that ventilation frequency increased significantly at escape depths >200 m, and was proportional to escape depth. As an indicator of airway obstruction, EF50 increased significantly at escape depths below 200 m, and the rate of increase increased with escape depth. By contrast, the Tr value decreased significantly at escape depths below 200 m, and the rate of decrease increased with escape depth. Penh, which reflects airway resistance, increased significantly after 200-m and 250-m escape, but the increase at 250 m was less than that at 200 m. PIF and PEF, which reflect the conductivity index, were significantly increased after 200-m escape, and more so after 250-m escape. Regarding lung volume, VT increased significantly after 200-m escape, but not after 220-m and 250-m escape. For cumulative lung volume, AV increased significantly after 200-m and 220-m escape but decreased significantly after 250-m escape. Te decreased significantly after 200-m escape, and the rate of decrease increased with escape depth. Ti decreased after 200-m escape and decreased further after 250-m escape. Finally, Rpef decreased significantly after 250-m escape, and showed non-significant decreases after 150-m, 200-m, and 220-m escape.

### Effect of escape depth on acid–base status

The changes in acid–base status are shown in Figure 3C. The pH values of the 150-m, 200-m, and 220-m escape groups were within the normal range (7.35–7.45) and were similar to the control group; however, the pH of the 250-m escape group was significantly lower than that of the control group (7.26 ± 0.10 *vs.* 7.38 ± 0.03; *p* = 0.019). The oxygen partial pressure (PO_2_) of the 150-m escape group was in the normal range (106.17 ± 8.27 mmHg) but decreased markedly in the 200-m (84.47 ± 14.10 mmHg), 220-m (76.38 ± 12.56 mmHg), and 250-m (76.5 ± 29.77 mmHg) escape groups; the PO_2_ values of the 220-m and 250-m escape groups were lower than the normal value (80 mmHg). Compared to the control group, the PO_2_ of all escape groups decreased significantly, and the magnitude of decrease was proportional to the escape depth. The carbon dioxide pressure (PCO_2_) was within the normal range in all escape groups and did not differ significantly compared with the control group. The HCO_3_^−^ results were consistent with those of PCO_2_—there was no significant change in the escape groups. The anion gap (AG) values of all escape groups were within the normal range, but that of the 250-m escape group was significantly higher than the control group (13.57 ± 3.47 mmol/L *vs.* 6.53 ± 2.58 mmol/L; *p* = 0.003). The actual base excess (AE) values of the escape group were also within the normal range, but the values of the 200-m, 220-m, and 250-m escape groups were significantly lower than that of the control group (−0.52 ± 1.26 mmol/L, −0.56 ± 1.48 mmol/L, and −2.02 ± 2.21 mmol/L *vs.* 1.26 ± 0.90 mmol/L; *p <* 0.05). The magnitude of decrease was consistent with the escape depth.

### Effect of escape depth on blood cell counts

**FIGURE 4:**
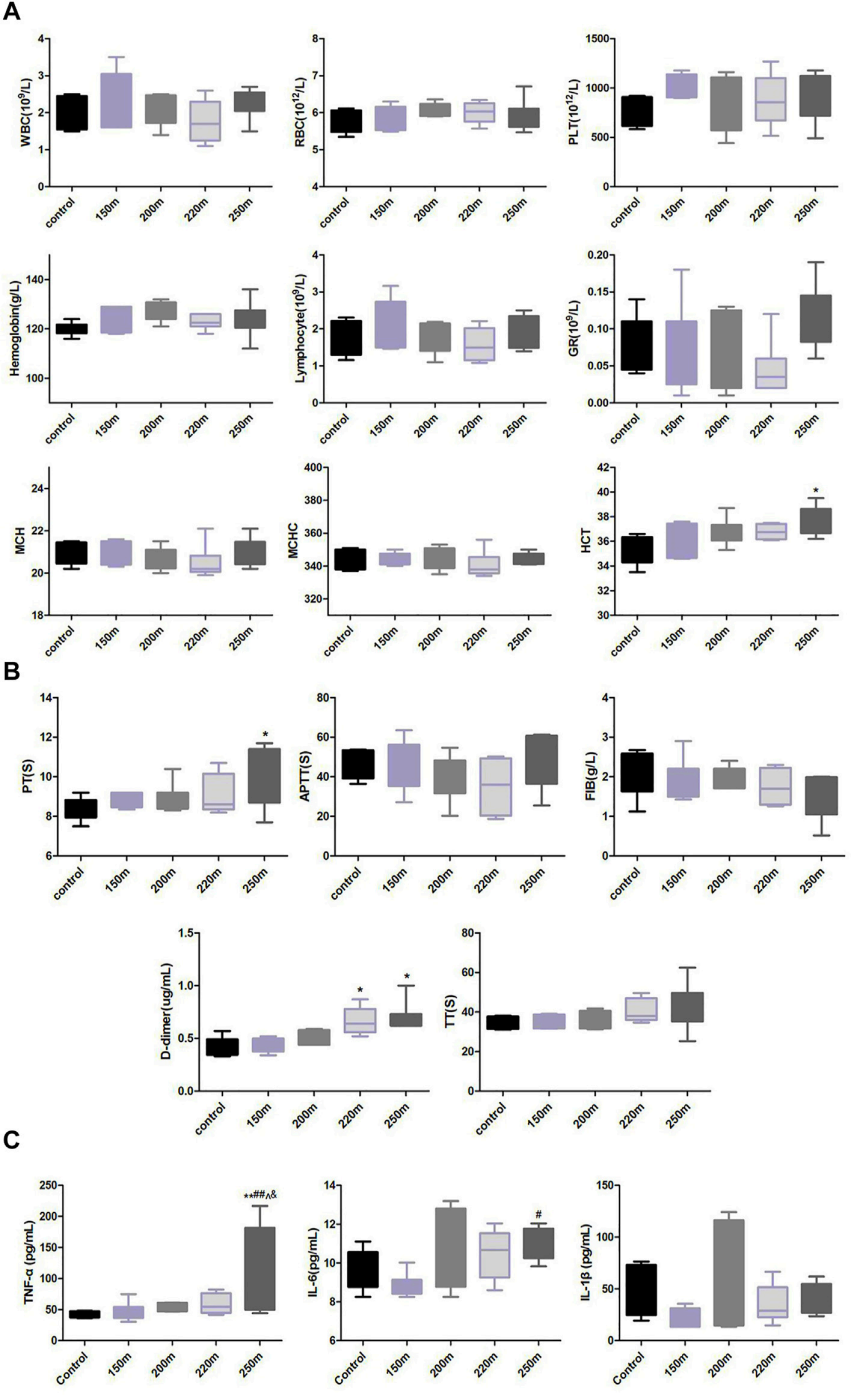
Effect of depth of simulated submarine tower escape on peripheral blood cells **(A)**, coagulation and fibrinolysis indexes **(B)**, and lung levels of proinflammatory cytokines **(C)**. One-way ANOVA was performed based on the normality of the distribution by Kolmogorov–Smirnov test. Results are the minimum to maximum values (*n* = 5–8 per group). *p < 0.05, **p < 0.01 compared to the control; ^##^ *p* < 0.01 compared to 150-m escape; ^*p* < 0.05 compared to 200-m escape; ^&^ *p* < 0.05 compared to 220-m escape.

Compared to the control group, the hematocrit and hemoglobin levels and red blood cell count in the escape groups presented an increasing, albeit non-significant, trend (Figure 4A). The rats in the 250-m escape group had significantly higher homocysteine (HCT) values (37.64 ± 1.12% *vs.* 35.44 ± 1.21%; *p* = 0.76) than the control group. There were no significant changes in platelet, white blood cell, mean corpuscular hemoglobin, and mean corpuscular hemoglobin concentration values.

### Effect of escape depth on coagulation parameters

The D-dimer value increased with increasing escape depth (Figure 4B). Compared with the normal control group, the D-dimer values in the 220-m and 250-m escape groups increased significantly (0.67 ± 0.06 μg/mL, 0.69 ± 0.13 μg/mL *vs*. 0.42 ± 0.04 μg/mL; *p* < 0.05). The FIB values in all escape groups decreased non-significantly with increasing escape depth. The PT value increased with escape depth, and was significantly higher in the 250-m escape group than the control group (10.09 ± 1.03 s *vs*. 8.34 ± 0.24 s; *p* < 0.05). The TT value increased, albeit non-significantly, with escape depth. The aPTT value first decreased with the escape depth to a nadir at 220 m, and increased at 250 m, albeit non-significantly.

### Effect of escape depth on proinflammatory cytokine levels

The levels of IL-6 and TNF-α were similar in the 150-m escape group and the control group. At escape depths >200 m, the IL-6 and TNF-α levels increased with escape depth (Figure 4C), and were significantly higher in in the 250-m escape group than the control group (IL-6: 10.97 ± 0.82 pg/mL *vs.* 9.52 ± 1.04 pg/mL; *p* = 0.023; TNF-α: 121.30 ± 71.55 pg/mL *vs.* 43.03 ± 5.31 pg/mL; *p* < 0.05). The IL-1β level did not change significantly in any of the escape groups.

## Discussion

Submarine escape by free ascent enables survivor egress from a DISSUB. The theory of submarine escape by free ascent is that through rapid pressurization, a small amount of inhaled inert gas will not produce bubbles during rapid decompression. However, human physiology limits the depth of escape, principally as a result of DCI. The effect of depth escape and the safe depth range for escape are unknown.

Prior animal studies have shown that decompression damage caused by submarine escape by free ascent is mainly to the heart and lungs, accompanied by changes in the coagulation and fibrinolysis system and subsequent activation of the inflammatory system. Therefore, we evaluated the effect of escape depth on ECG parameters, pulmonary function indexes, the myocardial enzyme spectrum, blood gas parameters, coagulation and fibrinolysis indicators, and inflammatory factors.

At an escape depth of 150 m, there was no significant difference in the indexes compared to before escape. At an escape depth of 200 m, increased f and MV values indicated an increased respiratory rate and MV; significantly increased EF50 and Penh, as well as shortened Tr time, indicated increased airway resistance, and the decrease in Rpef indicated a small airway obstruction and decreased gas diffusion.

Blood gas values were the earliest indicators of change. The PO_2_ and alkali remaining were significantly reduced at 200 m, and more so at 220 m and 250 m. Indicators of heart damage and changes in the coagulation and fibrinolysis system were also sensitive to escape depth. The myocardial enzyme spectrum and D-dimer values increased significantly at 220 m. Inflammatory factor, HCT, and hemoglobin levels and the red blood cell count increased with escape depth. Compared with the control group, the IL-6, TNF-α, and HCT levels in the 250-m escape group were significantly increased. The results suggest that 200 m is the threshold depth for safe escape in this animal model.

Unsafe submarine escapes cause more severe DCI than diving. DCI caused by unsafe submarine escape is mainly the result of cardiopulmonary injury, manifesting as pulmonary hypertension, acute pulmonary edema, and right heart failure. Indeed, pulmonary edema (Bao et al., 2015; Wang et al., 2018), hemorrhage and myocardial injury (Yiqun et al., 2017), inflammation (Wang et al., 2018), platelet activation (Bao et al., 2015), and coagulation–fibrinolysis system activation are critical phenomena in DCI in submarine escape. This is consistent with our results. With increasing escape depth, the respiratory system changed first, followed by cardiac damage, and finally changes of inflammatory factors and blood indicators. The increases of blood indexes such as HCT and red blood cells may be caused by a decrease of systemic blood volume, suggesting increased vascular endothelial permeability. HCT increased significantly at 250 m escape depth. Circulating bubbles lead to capillary leak syndrome, extravasation of plasma, and hemoconcentration. In animals, hemoconcentration has been linked to a poor prognosis. HCT values of ≥48 increase the risk of severe and persistent neurological deficits in divers with DCI (Boussuges et al., 1996).

Severe DCI from diving is more commonly manifests as nervous system injury because air bubbles in the veins enter the arterial system through abnormal openings between the arteries and veins, such as the patent foramen ovale, leading to infarction of peripheral and central nervous tissue (Lafère et al., 2017). The DCI caused by submarine escape involves generation of a large number of air bubbles during rapid decompression after rapid pressurization to great depth. These air bubbles return to the right heart and subsequently remain in the pulmonary circulation, resulting in pulmonary infarction (Yiqun et al., 2017). Gas embolism in the pulmonary vascular bed can increase pulmonary arterial pressure, pulmonary vascular resistance, and right-heart load. Pulmonary gas embolism produces a transient partial obstruction in the pulmonary circulation and the performance of the right ventricle determines the maximum degree of embolization compatible with sufficient circulation (Verstappen et al., 1977). Decompression injury varied greatly among the rats after simulated submarine escape, likely due to the compensatory capacity of the right ventricle. Individuals with a well-compensated right ventricle will survive, while those with poor compensation will die within 2–3 min of decompression.

Pulmonary air embolism results in increased pulmonary vascular resistance, reduced cardiac output, systemic hypoxia, and hypercapnia (Oyama and Spencer 1971; Verstappen et al., 1977; Cockett et al., 1979). With increasing escape depth, rats gradually developed hypoxia and hypercapnia. The pH of the 250-m escape group was significantly lower than normal. The pH and PO_2_ values decreased with increasing escape depth. The PO_2_ value of the 220-m and 250-m escape groups were lower than normal, indicating hypoxia and acidemia. Elevated AG and decreased AE also suggested acidosis. Therefore, hypoxia is the earliest manifestation of decompression injury caused by submarine escape. The PO_2_ value was normal at an escape depth of 150 m, and decreased significantly at 200 m, indicating that escape is unsafe at depths >200 m.

Decompression stress affects the blood coagulation system. Microbubbles affect clotting by activating coagulation and inducing platelet aggregation (Barak and Katz, 2005). The bubble surface acts as a foreign material and activates the coagulation cascade (Pontier et al., 2011). The microbubble gas–blood interface adsorbs macromolecules in blood. Adsorption causes conformational changes in the molecule, such as unfolding, and exposes regions of the protein that trigger blood clotting (Philp et al., 1972). Decompression injury can decrease circulating platelets and increase platelet–leukocyte complexes. Plasma D-dimer can be used to predict severe neurological DCI in scuba divers (Gempp et al., 2012). In this study, the D-dimer level changed significantly at escape depths ≥220 m. The PT value significantly increased at 250 m. The TT and FIB values changed with increasing escape depth. These results suggest that decompression stress affects the coagulation and fibrinolysis systems at escape depths ≥220 m.

Inflammatory response is an important manifestation of decompression injury. Air bubbles cause vascular endothelial damage, leading to adsorption of adhesion factors, activation of complement, and triggering of inflammation (Yu et al., 2021; Schirato et al., 2022). The levels of inflammatory factors showed an upward trend with escape depth. At an escape depth of 250 m, the IL-6 and TNF-α levels were significantly increased compared with the control group. Therefore, decompression injury can activate the inflammatory system. Moreover, at an escape depth reaching 250 m, systemic inflammatory activation appears.

An increase in pulmonary artery (PA) pressure is the most prominent indicator of pulmonary air embolism. PA pressure rises more rapidly with increasing rate of air entering the pulmonary circulation. This is followed by a rapid drop in end-tidal carbon dioxide and oxygen saturation values. ECG can detect a variety of changes, including tachyarrhythmias, varying degrees of atrioventricular block, right ventricular strain, and ST-segment changes. Unfortunately, due to technical problems, PA pressure and right ventricular output were not measured in this study. The ECG parameters changed with increasing escape depth. Increased R waves appeared with increasing escape depth, suggesting ventricular hypervoltage. Small Q waves appeared at an escape depth of 200 m. At an escape depth of 220 m, pathological Q waves appeared in multiple leads, suggesting myocardial ischemia.

Gas exchange abnormalities associated with pulmonary air embolism include decreased PO_2_, increased PCO_2_, and an increase in the alveolar–arterial oxygen difference (Hlastala et al., 1979). Ventilation–perfusion (VA/Q) inhomogeneity is the primary cause of these gas exchange abnormalities. When VA/Q is out of balance, gas diffusion is hindered, resulting in hypoxia and hypercapnia. The body compensates by increasing the respiratory rate to maintain MV. In this study, an escape depth >200 m significantly increased ventilation frequency, airway obstruction, and small airway obstruction and decreased gas diffusion.

## Conclusion

Safe at 150 m in this animal model, but there is a risk of harm at depths >200 m. The main manifestations are pulmonary ventilation and gas diffusion dysfunction, hypoxemia, myocardial ischemia, and activation of the fibrinolytic and inflammatory systems. Respiratory system changes show the greatest sensitivity to escape depth, and the performance of the right ventricle is a key determinant of prognosis.

## Limitations

The limitation of this study is that the physiological structures of rats and humans are too different to be directly analogized. We also did a lot of goat experiments in the follow-up experiments. Compared with rats, goats are closer to humans in body weight, and body weight is an important factor affecting safe escape. The Royal Navy has used goats to define DCL risks following simulated submarine escape (Blogg et al., 2003; Seddon et al., 2014). Six goats were used for the escape depths of 150 m, 200 m, and 250 m respectively. The results showed that goats in the 150 m and 200 m escape depth groups did not have obvious symptoms of decompression sickness, and the heart bubbles detected by Doppler were all grade 1. Goats in the 250 m escape depth group had obvious decompression sickness symptoms, including nervous system symptoms such as hemiplegia and gastrointestinal barotrauma. The data of goats also suggest that escape at > 200 m can be harmful. However, animal experiments cannot be directly analogized to humans. More experiments are needed to verify the maximum escape depth that humans can achieve.

## Data Availability

The original contributions presented in the study are included in the article/Supplementary material, further inquiries can be directed to the corresponding authors.

## References

[B1] Bao X. C. Chen H. Fang Y. Q. Yuan H. R. You P. Ma J. (2015). Clopidogrel reduces the inflammatory response of lung in a rat model of decompression sickness. Respir. Physiology Neurobiol. 211, 9–16. 10.1016/j.resp.2015.02.003 25784626

[B2] Barak M. Katz Y. (2005). Microbubbles. Chest 128, 2918–2932. 10.1378/chest.128.4.2918 16236969

[B19] Blogg S. L. Gennser M. Loveman G. A. Seddon F. M. Thacker J. C. White M. G. (2003). The effect of breathing hyperoxic gas during simulated submarine escape on venous gas emboli and decompression illness. Undersea Hyperb. Med. 30, 163–174. 14620096

[B3] Boussuges A. Blanc P. Molenat F. Bergmann E. Sainty J. M. (1996). Haemoconcentration in neurological decompression illness. Int. J. Sports Med. 17, 351–355. 10.1055/s-2007-972859 8858406

[B4] Cockett A. T. Pauley S. M. Zehl D. N. Pilmanis A. A. Cockett W. S. (1979). Pathophysiology of bends and decompression sickness. Arch. Surg. 114, 296–301. 10.1001/archsurg.1979.01370270066011 435035

[B5] Donald K. W. (1979). Submarine escape breathing air. A review and analysis of animal and human experiments by the Royal Navy. Bull. Eur. Physiopathol. Respir. 15, 739–754. 389328

[B6] Gempp E. Morin J. Louge P. Blatteau J. E. (2012). Reliability of plasma D-dimers for predicting severe neurological decompression sickness in scuba divers. Aviat. space Environ. Med. 83, 771–775. 10.3357/asem.3323.2012 22872991

[B7] Hlastala M. P. Thomas Robertson H. T. Ross B. K. (1979). Gas exchange abnormalities produced by venous gas emboli. Respir. Physiol. 36, 1–17. 10.1016/0034-5687(79)90011-2 217052

[B8] Lafère P. Balestra C. Caers D. Germonpré P. (2017). Patent foramen ovale (PFO), personality traits, and iterative decompression sickness. Retrospective analysis of 209 cases. Front. Psychol. 8, 1328. 28824507 10.3389/fpsyg.2017.01328 PMC5539185

[B9] Oyama Y. Spencer.P M. P. (1971). Cardiopulmonary effects of intravenous gas embolism; with special reference to fate of intravascular gas bubbles. Jpn. Circ. J. 35, 1541–1549. 10.1253/jcj.35.12_1541 5172474

[B10] Philp R. B. Inwood M. J. Warren B. A. (1972). Interactions between gas bubbles and components of the blood: Implications in decompression sickness. Aerosp. Med. 43, 946–953. 4116740

[B11] Pontier J. M. Vallée N. Ignatescu M. Bourdon L. (2011). Pharmacological intervention against bubble-induced platelet aggregation in a rat model of decompression sickness. J. Appl. Physiology 110, 724–729. 10.1152/japplphysiol.00230.2010 PMC3069627 21212250

[B20] Seddon F. M. Thacker J. C. Fisher A. S. Jurd K. M. White M. G. Loveman G. A. (2014). Decompression illness in goats following simulated submarine escape: 1993–2006. Undersea Hyperb. Med. 41, 301–306. 25109083

[B12] Schirato S. R. Silva V. Silva K. Iadocicco A. Marroni M. Pieri D. (2022). Post-decompression bubble and inflammation interactions: A non-extensive dynamical system model. Uhm 49, 207–226. 10.22462/03.04.2022.6 35580488

[B13] Verstappen F. T. Bernards J. A. Kreuzer F. (1977). Effects of pulmonary gas embolism on circulation and respiration in the dog. Pflugers Arch. 368, 89–96. 10.1007/bf01063459 558602

[B14] Wang H. T. Fang Y. Q. Bao X. C. Yuan H. R. Ma J. Wang F. F. (2015a). Expression changes of TNF-α, IL-1β and IL-6 in the rat lung of decompression sickness induced by fast buoyancy ascent escape. Undersea Hyperb. Med. 42, 23–31. 26094301

[B15] Wang H. T. Fang Y. Q. Fang P. Yu X. C. Bao K. C. Li J. (2018). PDTC ameliorates decompression induced-lung injury caused by unsafe fast buoyancy ascent escape via inhibition of NF-κB pathway. Uhm 45, 351–362. 10.22462/05.06.2018.10 30028921

[B16] Wang H. T. Fang Y. Q. You P. Bao X. C. Yuan H. R. Ma J. (2015b). Expression changes of inflammatory factors in the rat lung of decompression sickness induced by fast buoyancy ascent escape. Undersea Hyperb. Med. 42, 15–22. 26094300

[B17] Yiqun F. Pu Y. Pu W. Haitao B. Xiaochen M. Jun Z. (2017). Metabonomic potential plasma biomarkers in abnormal fast buoyancy ascent escape-induced decompression sickness model and the protective effects of pyrrolidine dithiocarbamic acid. Uhm 44, 109–119. 10.22462/3.4.2017.4 28777901

[B18] Yu X. Xu J. Liu W. Zhang Z. He C. Xu W. (2021). Protective effects of pulmonary surfactant on decompression sickness in rats. J. Appl. Physiology 130, 400–407. 10.1152/japplphysiol.00807.2020 33270509

